# Construction Notes

**Published:** 1893-05-20

**Authors:** 


### CONSTRUCTION NOTES.

The Oldham Infirmary.—During the past year new heating arrangements have been effected at this institution by means of Messrs. Langfield and Co.'s aparatus in connection with Tobin's tubes. The new arrangements have greatly added to the comfort of the patients and the staff.

"The Children's Cot" at the Morecambe Infirmary.—The Committee of the Morecambe Infirmary are devoting the £400, which was the result of a sale of work by a number of Lancaster ladies, to the providing of a bed in the children's ward, which is to be named "The Children's Cot."

Convalescent Home at Bewdley.—The commencement of April saw the opening of the "Odd Fellows' Convalescent Home at Bewdley." The home, which is intended to benefit members of lodges in Midland Counties within a radius of 30 miles of Birmingham, is situated on a hill on the western side of the Severn. The site has been purchased and the home furnished at a cost of about £1,500. At present it will accommodate 20 convalescents, but should occasion require a still larger number may be received. Ample provision for pastimes, indoor and outdoor, has been considered. The home is admirably planned and fitted, and will prove a great boon to the surrounding district.

